# Feasibility Analysis of Tetracycline Degradation in Water by O_3_/PMS/FeMoBC Process

**DOI:** 10.3390/molecules30244810

**Published:** 2025-12-17

**Authors:** Xuemei Li, Qingpo Li, Jian Wang, Zheng Wu, Shengnan Li, Hai Lu

**Affiliations:** 1 College of Visual Arts, Changchun Sci-Tech University, Changchun 130600, China; 100464@cstu.edu.cn; 2 Key Laboratory of Songliao Aquatic Environment, Ministry of Education, Jilin Jianzhu University, Changchun 130118, China; liqingpo@student.jlju.edu.cn (Q.L.); lishengnan@neau.edu.cn (S.L.); 3 Basic Research Department, Changchun Sci-Tech University, Changchun 130600, China; 100495@cstu.edu.cn (J.W.); 100817@cstu.edu.cn (Z.W.)

**Keywords:** Fe–Mo modified biochar, ozone, peroxymonosulfate, tetracycline degradation, toxicological assessment

## Abstract

In this study, the feasibility of tetracycline (TC) degradation in water using Fe–Mo co–supported biochar (FeMoBC) as catalyst combined with ozone and peroxymonosulfate (O_3_/PMS) system is discussed. The experiment showed that the mineralization rate of TC by O_3_/PMS/FeMoBC process reached 60.1% within 60 min, which was significantly higher than the treatment effect of O_3_ or O_3_/PMS system alone. Meanwhile, this process showed higher degradation efficiency under the background of raw water, and the loss of FeMoBC cycle attenuation performance was small. Twelve intermediates in the degradation of TC were identified by ultra-high performance liquid chromatography–tandem mass spectrometry (UPLC–MS/MS), and the possible degradation paths were inferred by quantum chemical calculation. In addition, the toxicity of intermediate products was evaluated by ecological structure–activity relationships (ECOSAR) and toxicity estimation software tool (T.E.S.T.) software, and the results showed that with the degradation of TC, its toxicity showed a downward trend as a whole. Therefore, this study confirmed that O_3_/PMS/FeMoBC had high efficiency in degrading TC in actual water, which provided a new idea for the treatment of high concentration organic wastewater.

## 1. Introduction

With the development of science and technology and industry, antibiotics are produced in large quantities and widely used, which leads to the detection of antibiotics and their transformants in the water environment of more and more countries and regions. Antibiotics have a low absorption rate in human and animals, and are eventually excreted in feces and urine. Antibiotics and their conversion products (conjugated state, oxidation products, hydrolysis products, etc.) are released into surface water and groundwater. Other sources of antibiotics include wastewater from hospitals and pharmaceutical factories, etc. [1].

Among the commonly used antibiotics, tetracycline antibiotics are widely used in the world because of their low price. Its types mainly include chlortetracycline, oxytetracycline, tetracycline and semi-synthetic derivatives such as methotrexate, doxycycline and minocycline, etc. The solid–liquid partition coefficient (K_d_ value) of tetracycline antibiotics is 4.2 × 10^4^~1.0^3^ × 10^5^ mg/kg, which is higher than other antibiotics. Therefore, tetracycline antibiotics entering the environment are more likely to be adsorbed and accumulated in the soil, thus destroying the microbial community in the soil environment, inhibiting the growth of beneficial microorganisms in the soil and accelerating the spread of resistance genes in the water environment [2]. With surface runoff and leaching, tetracyclic antibiotics adsorbed in soil can migrate to groundwater and surface water environments. This kind of pollution contributes to the spread of resistance genes and becomes one of the long-term risk factors of aquatic ecosystems [3]. Up to now, the removal of TC by biological methods, physical adsorption and advanced oxidation has been widely reported. At present, the mainstream research direction is to catalyze oxidants through materials to achieve higher utilization and degradation efficiency of oxidants.

After ozone/peroxymonosulfate (O_3_/PMS) combined technology was put forward, atrazine was efficiently degraded in a short time [4]. This process can simultaneously produce ^•^OH and SO_4_^•−^. Compared with ^•^OH, SO_4_^•−^ has a higher redox potential (E^0^ = 2.5–3.1 eV), a longer half–life (30–40 μs) and a wider pH environment, which has obvious advantages compared with the traditional advanced oxidation process. SO_4_^•−^ in O_3_/PMS process is mainly provided by PMS. SO_4_^•−^ has a high redox potential, which is equivalent to that of ^•^OH (2.5–3.1 eV) under neutral conditions, but slightly higher than ^•^OH (1.9 eV) under acidic conditions [5,6]. In a conventional environment, the decomposition rate of persulfate anion (S_2_O_8_^2−^) to produce SO_4_^•−^ is slow, so the effect of PMS used alone is not significant [7,8]. Therefore, many methods of activating PMS were derived, including thermal decomposition, ultrasonic, ultraviolet irradiation, alkali activation, laser flash activation and transition metal catalysis.

Based on the above research results, more researchers try to degrade refractory organic wastewater, such as antibiotic wastewater, by catalyzing the O_3_/PMS system with cheap metal materials. The role of catalyst in this process was mainly reflected in improving reaction efficiency, reducing energy consumption [9], reducing reaction byproducts [10], enhancing reaction selectivity, promoting the moderation of reaction conditions, and sustainability and recycling [11,12].

Nano-zero-valent iron (nZVI) is a kind of metal nano-material with high reducibility and catalytic performance, which can undergo electron transfer reaction with persulfate (PS) and promote its decomposition to generate free radicals [13]. Loading nZVI on BC can overcome the shortcomings of nZVI, such as easy aggregation, oxidation and inactivation, and improve its dispersibility, stability and utilization rate. Meanwhile, it can also use the adsorption of BC to enhance the enrichment and contact of TC in water, thus improving the degradation efficiency [14,15]. Molybdenum (Mo) is a typical transition metal. The electronic configuration of the d orbital of Mo is flexible, which makes it have many valence states (Mo^0^, Mo(II), Mo(III), Mo(IV), Mo(V) and Mo (VI)), and it has great advantages in many redox reactions [16]. In addition, due to the differences in local environment and crystal structure, Mo-based compounds may have different physical or chemical properties even if they have the same composition, thus showing different catalytic activation properties [17]. In recent years, there have been a lot of studies on using Mo-based materials as catalysts or cocatalysts in the field of heterogeneous catalytic oxidation degradation of organic pollutants [18].

It is a common use of catalysts to make up for the defects of the original process by composite materials, such as controlling bromate, improving reaction rate and reducing energy consumption [19]. For bimetallic catalysts with iron as the main element, their electronic structure and geometric structure are different from those of corresponding monometallic catalysts. However, the correlation between reactivity and structural factors is still unknown. Some researchers believe that in the bimetallic catalysts reaction system, another metal element usually acts as an electron donor, and the main reaction is the coupling of iron and oxidant (Fenton and Fenton-like) [20,21].

Therefore, in this study, iron ions and molybdenum ions with good activation efficiency for both O_3_ and PMS were used as activation metals, and they were loaded on biochar (BC) made from agricultural waste corn stalks to realize their activation. In order to solve the problem of ion leaching of bimetallic substrate in water and keep the metal ions in low valence state after calcination, the composite material was prepared by wet impregnation pyrolysis with Fe/Mo mixed salt as raw material. In this process, bimetallic oxides were loaded on the pores and surfaces of BC, and then the metal biochar composite material, namely FeMoBC, was prepared. When the material was applied to O_3_/PMS process, O_3_/PMS/FeMoBC system was formed. In this way, the existence of bimetal would improve the electron transfer rate in the reaction system, and then accelerate the catalytic reaction rate of the system. In this way, it was expected to realize the effective treatment of refractory organic wastewater. In addition, the carbon material in this process could also inhibit the formation of bromate [19].

In order to effectively verify the practical feasibility of O_3_/PMS/FeMoBC process to degrade TC in water, this study compared the treatment effects of O_3_, O_3_/PMS and O_3_/PMS/FeMoBC processes on TC wastewater. In the experiment, the removal rate of total organic carbon (TOC), the treatment effect of raw water sample and the circulation attenuation of catalyst in O_3_/PMS/FeMoBC process were investigated in detail. In addition, the quantum chemical calculation of molecular structure, the analysis of intermediate products in the process of TC degradation and the degradation mechanism of TC were carried out. Finally, the toxicity analysis of the above products was carried out by ECOSAR v 2.2 (U.S. Environmental Protection Agency, Washington, DC, USA) and T.E.S.T. v 5.1 (U.S. Environmental Protection Agency, Washington, DC, USA) software.

## 2. Materials and Methods

### 2.1. Materials and Reagents

Reagents used in the experiment are shown in Table 1. Tetracycline hydrochloride (TC), formic acid, L–histidine and p–benzoquinone (p–BQ), purity > 99%, were purchased from Shanghai McLean Biochemical Technology Co., Ltd. (Shanghai, China). Potassium peroxymonosulfate and methanol, with excellent purity, were purchased from Sigma-Aldrich Company (St. Louis, MI, USA). Reagents such as ferric chloride hexahydrate, sodium molybdate, sodium thiosulfate, sodium chloride, sodium nitrate, tert-butanol and sodium bicarbonate were all analytically pure and purchased from China Sinopharm Group Chemical Reagents Co., Ltd. (Shanghai, China). 

All the solutions in the experiment were prepared by ultra-pure water machine (Molelement elemental type 1820a water system, Molecular scientific instrument limited company, Shanghai, China). Raw water samples were collected from the fine grid of Southeast Wastewater Treatment Plant in Changchun City, Jilin Province, China. After being retrieved, they were allowed to stand for 24 h, filtered twice with 50 μm qualitative filter paper, and stood in the dark for later use.

The experimental setup, preparation process of FeMoBC materials and the process of the degradation experiments can be found in other papers published by the authors [22].

### 2.2. Analysis and Detection Methods

Thermo Fisher UltiMate 3000 Ultra Performance Liquid Chromatography–Tandem Mass Spectrometry (UPLC–MS/MS, Thermo Fisher Scientific Inc., Waltham, MA, USA) was used to analyze TC intermediates. The detector was an ultraviolet detector, and the separation was carried out on a Poroshell EC-C18 chromatographic column (specification: 2.1 × 100 nm, particle size: 2.7 nm, Agilent Technologies, Santa Clara, CA, USA). The composition of mobile phase was 0.1% formic acid: acetonitrile = 85:15 (volume ratio), the flow rate was 20 μL/min, and the injection volume was 1000 μL. Electrospray ionization source (ESI) was used in the mass spectrometry, and the ion source voltage was set to 30 V. Positive ion mode was used for full scanning, and the scanning range of mass-to-charge ratio was 50–500 *m*/*z*. Aqualog 800-C absorption three-dimensional fluorescence spectrometer of Horiba Scientific Company (Shanghai, China) was used to analyze the changes in water samples before and after the experimental reaction of raw water. Shimadzu TOC-V_CPH_ total organic carbon analyzer (Shimadzu Corporation, Kyoto, Japan) was used to analyze the TOC changes in raw water before and after the experimental reaction. The water quality index was measured by 725N ultraviolet-visible spectrophotometer of Shanghai Yidian (Group) Co., Ltd., Shanghai, China.

### 2.3. Overview of Characterization Results of FeMoBC Materials

SEM images of FeMoBC showed that its surface was rough and irregular, and many metal particles were evenly distributed on the surface and pores of BC, which promoted the electron transfer of catalyst in the oxidation experiment. Meanwhile, through EDS analysis, it was clear that there were elements such as carbon, oxygen, iron and molybdenum in FeMoBC materials, and their mass proportions were 41.56%, 26.31%, 18.82% and 13.31%, respectively. The analysis confirmed that both Fe and Mo were loaded on the surface and pores of BC. The FTIR spectra of FeMoBC produced new peaks, which were mainly caused by the formation of new surface functional groups after Fe/Mo co-loading. Two new absorption bands appeared at about 450 cm^−1^ and 558 cm^−1^, which were generally considered as FeO, while the two new absorption bands at 805 and 932 cm^−1^ were MoO stretching vibration peaks. Compared with FeMoBC (new), the intensity of some peaks of FeMoBC (used) was slightly attenuated, indicating that some functional groups were consumed in the catalytic reaction, so the catalytic reaction was mainly concentrated on the catalyst surface. The above phenomenon showed that many oxygen-containing functional groups of Mo and Fe were successfully loaded on FeMoBC. Through the comparison of surface structure characteristics, it was found that the specific surface area of BC and FeMoBC was 28.8771 and 80.3445 m^2^/g, the micropore area was 18.0232 and 44.5264 m^2^/g, the total pore volume was 0.091465 and 0.094498 cm^3^/g, and the average pore size was 2.4159 and 4.7046 nm, respectively. The above results showed that compared with BC, the specific surface area, micropore area, total pore volume and average pore size of FeMoBC modified by Fe/Mo metal had significantly increased. This phenomenon was caused by the successful loading of Fe/Mo metal nanoparticles on the surface of BC [22].

In addition, XRD spectrum showed that BC had a typical planar graphite amorphous carbon structure (JCPDS 41–1487), and its diffraction peak was at 2θ = 24.5°, which usually had strong supporting performance of metal carrier. There was a diffraction peak at 26.16° in the XRD diffraction pattern, and weak peaks at 2θ values of 26.97°, 31.62° and 33.52°. Therefore, according to the PDF standard card (PDF#89-2367), the peak belonged to FeMoO_4_. Through the analysis of the above two crystal phases by JADE 9.0 software, it was concluded that the proportions of Fe_2_Mo_3_O_8_ and FeMoO_4_ were 26.3% and 73.7%, respectively. FeMoBC (new) and FeMoBC (used) were characterized by XPS spectrum analysis. The results showed that the five peaks at 285.08, 531.08, 712.08, 398.08 and 233.08 eV were higher in the full spectrum of the catalyst, corresponding to C1 s, O1 s, Fe 2P, N1 s and Mo 3d respectively. In addition, the peak intensities of elements C, O, Fe, N and Mo in different orbits changed slightly after the reaction, indicating that these substances all participated in the reaction. C1 s, Fe 2p and Mo 3d diagrams showed the changes in surface active sites and chemical composition of elements such as C, Fe and Mo in FeMoBC catalyst before and after the reaction. This was consistent with the detection results of XRD, which further showed the successful synthesis of FeMoBC [22].

## 3. Results

### 3.1. TOC Removal Rate of TC Degraded by O_3_, O_3_/PMS and O_3_/PMS/FeMoBC Processes

Under the conditions of water phase temperature of 25 ± 1 °C, [PMS]_0_ = 30 μM, gaseous ozone concentration of 3.6 mg/L, solution pH of 6.8 ± 0.1, composite material dosage of 200 mg/L and [TC]_0_ = 0.03 mM, experiments were carried out to investigate the TOC removal rates of TC in water by O_3_, O_3_/PMS and O_3_/PMS/FeMoBC. During the experiment, samples were taken at 10, 20, 30, 40, 50 and 60 min, respectively.

**Figure 1:**
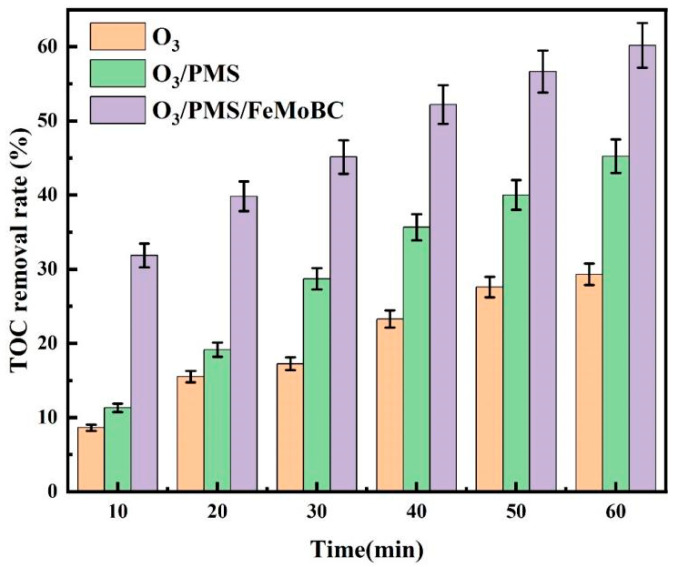
TOC removal rates of TC degradation by O_3_, O_3_/PMS and O_3_/PMS/FeMoBC. Reaction conditions: water phase temperature of 25 ± 1 °C, [PMS]_0_ = 30 μM, gaseous ozone concentration of 3.6 mg/L, solution pH of 6.8 ± 0.1, FeMoBC dosage of 200 mg/L and [TC]_0_ = 0.03 mM.

The experimental results are shown in Figure 1. It can be seen from Figure 1 that the TOC removal rates of TC in O_3_ process were 8.62%, 15.517%, 17.241%, 23.276%, 27.586% and 29.310% at 10, 20, 30, 40, 50 and 60 min, respectively. In O_3_ process, the degradation rate of TC was about 90% in 20 min [22]. Therefore, combined with the numerical analysis of TOC removal rate, it is not difficult to see that O_3_ process had poor ability for deep degradation of TC. The mineralized part was less as CO_2_ and H_2_O, while the non-mineralized part was mainly an intermediate product in the form of macromolecular organic matter.

Meanwhile, the TC degradation rate by O_3_/PMS could reach over 99% in 20 min [22], but the TOC removal rate was only 19.1%. When it reached 60 min, the TOC removal rate of O_3_/PMS system could reach 45.2%; therefore, its mineralization ability is stronger than that of O_3_ process. In addition, the increment of mineralization rate decreased slightly with the increase in reaction time, but it was better than O_3_ process. There were two main reasons for this phenomenon. First, with the continuous reaction, the concentration of PMS in the reaction system kept decreasing, which led to the formation of SO_4_^•−^ mainly concentrated in the first 20 min. In addition, when SO_4_^•−^ was converted by ^•^OH, its continuous combination with the target pollutants led to the continuous decrease in its concentration [23]. Therefore, the subsequent mineralization reaction was mainly completed by ^•^OH, which led to the gradual decline of mineralization efficiency of O_3_/PMS process after 20 min. Secondly, the accumulation of refractory intermediates might occur during the reaction [24]. In the subsequent reaction, the energy provided by active substances was less than the electrochemical potential energy of intermediate products, which was not enough to decompose the latter, thus leading to the continuous accumulation of intermediate products, which further affected the degradation rate of TOC [25].

In the TOC removal rate trend of O_3_/PMS/FeMoBC process, the TOC removal rate could reach 60.1% in 60 min, which was significantly higher than that of O_3_ and O_3_/PMS processes. The degradation rate of TC by O_3_/PMS/FeMoBC process reached about 97% in 10 min [22], while the TOC removal rate was only 31%. This was due to the direct adsorption of part of TC by FeMoBC material at the initial stage of the reaction, and then the catalyst improved the generation and utilization rate of free radicals and quickly mineralized part of TC [26]. The experimental results showed that the overall mineralization rate was higher in the O_3_/PMS/FeMoBC process. According to previous quenching experiments, compared with O_3_ and O_3_/PMS processes, the O_3_/PMS/FeMoBC system produced more ^•^OH and SO_4_^•−^, Meanwhile, ^•^O_2_ and ^1^O_2_ also existed in this system, so it had a better mineralization effect [22,27].

### 3.2. Degradation of TC by O_3_/PMS/FeMoBC Under the Background of Actual Water Body

**Figure 2:**
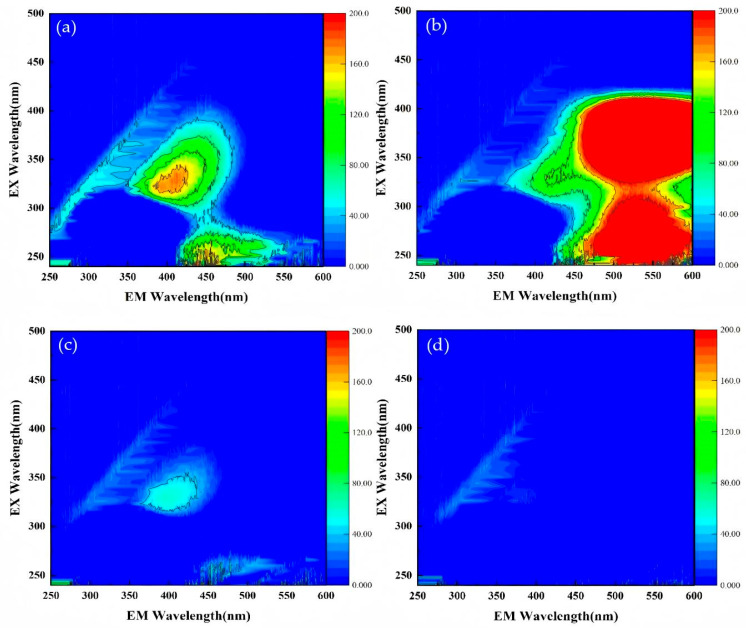
The 3D fluorescence spectra of water samples equipped with TC at the fine grid of Southeast Wastewater Treatment Plant. (**a**) untreated (without TC); (**b**) untreated (with TC); (**c**) treated for 20 min; (**d**) treated for 60 min.

**Figure 3:**
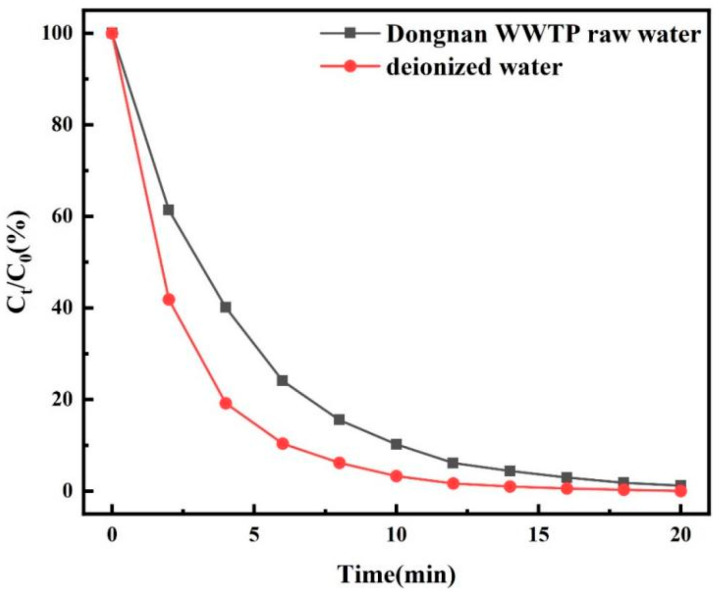
Effect of O_3_/PMS/FeMoBC on degradation of TC water samples under different water quality conditions.

In order to verify the ability of O_3_/PMS/FeMoBC process to degrade TC in complex water, the sewage samples at the fine grid of Southeast Wastewater Treatment Plant were tested. After standing and filtering, the raw water was prepared into 30 μM TC solution as a solvent. Meanwhile, the parameters of water samples were detected by ultraviolet spectrophotometer and three-dimensional fluorescence instrument, and then the degradation experiment of TC was carried out. The experimental results are shown in Table 1, Figure 2 and Figure 3.

As can be seen from Table 1, after degradation by the O_3_/PMS/FeMoBC process, the indexes such as COD, TN, NH_3_–N and TP in the solution decreased significantly, and the pH changed little before and after the reaction. Figure 2 shows that, according to the relationship between the types of organic substances and the positions of corresponding fluorescence peaks in Table 2, the organic substances in the original solution mainly existed between zones III, IV and V (240–400 nm/350–550 nm), and were judged as aromatic protein or fulvic acid and humic acids. After treated by O_3_/PMS/FeMoBC system for 20 min, the fluorescent substances in raw water samples decreased in a large area. After 60 min treatment, the fluorescence region almost disappeared, indicating that most fluorescent organic compounds had degraded below the detection limit at this time. With the progress of oxidative degradation, the content of these organic substances further decreased.

Figure 3 shows that in the O_3_/PMS/FeMoBC system, compared with deionized water samples, the time required to degrade the target pollutants dissolved in raw water samples to below 10% was increased by 6 min. It could be seen that the raw water samples with complex components had a certain impact on the degradation efficiency of O_3_/PMS/FeMoBC. The main reason was that there were many types of inorganic salts and other complex organic substances in raw water samples. In the process of oxidative degradation, they interfered with each other by quenching the active oxidized substances and robbing the active oxidized substances with the target pollution [28]. Nevertheless, the degradation rate of TC in water by O_3_/PMS/FeMoBC process could still reach 98.8%, which further showed that the system was applicable to the degradation of TC in the actual water background.

### 3.3. Effect of Catalyst Cycle Attenuation

Under the conditions of water phase temperature of 25 ± 1 °C, [PMS]_0_ = 30 μM, gaseous ozone concentration of 3.6 mg/L, [TC]_0_ = 0.03 mM, composite material dosage of 200 mg/L, rotor speed of 200 r/min and solution pH of 6.8 ± 0.1, the performance attenuation test of the catalyst was carried out five times. In the experiment, the catalyst was washed with deionized water after each use and then dried in a drying oven before being used again.

**Figure 4:**
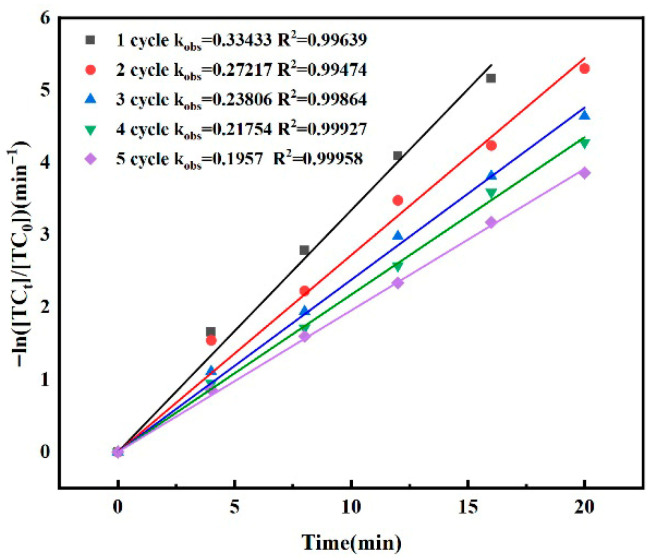
The degree of cyclic attenuation of catalyst. Reaction conditions: water phase temperature of 25 ± 1 °C, [PMS]_0_ = 30 μM, gaseous ozone concentration of 3.6 mg/L, [TC]_0_ = 0.03 mM, FeMoBC dosage of 200 mg/L, rotor speed of 200 r/min and solution pH of 6.8 ± 0.1.

The test results of catalyst performance attenuation are shown in Figure 4. The kinetic model of the five-cycle experiment was fitted to determine the removal rate of TC. After fitting, it was found that the reaction conformed to pseudo first-order kinetics and there was no adsorption. Equation (1) was the pseudo first-order kinetic model.

(1) $$\ln\left({TC}_{t}/{TC}_{0}\right)=-k_{obs}t$$

where *TC_t_* represented the concentration of TC at time *t*, *TC*_0_ was the initial concentration of TC, and *k_obs_* was the reaction rate.

It can be seen that after 1, 2, 3, 4 and 5 catalytic cycles, the pseudo first-order reaction rate (k_obs_ value) was 0.33433, 0.27217, 0.23806, 0.21754 and 0.1957 min^−1^, respectively. After being recycled five times, the catalytic efficiency of the catalyst decreased to some extent. This was because the active catalytic sites on the surface of the catalyst were gradually inactivated with the increase in reaction times, and the electron transfer ability was gradually reduced. However, the catalyst still had a certain catalytic ability after five cycles, which reflected the green and efficient performance of FeMoBC catalyst.

Based on the above experimental results and previous experimental conclusions about the degradation efficiency of TC in O_3_/PMS/FeMoBC, the roles of iron and molybdenum in the reaction could be clarified. That is, in the O_3_/PMS/FeMoBC reaction system, O_3_ could accept electrons from Fe^2+^ to generate ^•^OH and ^•^O_2_^−^ radicals. Ferrous ions in the solution then reacted with ^•^O_2_^−^ to generate H_2_O_2_, which in turn reacted with Fe^2+^ to generate more hydroxyl groups through Fenton-like reaction. Meanwhile, PMS was hydrolyzed into HSO_5_^−^ and SO_5_^−^ and further formed ^1^O_2_. With the progress of the reaction, the exposed Mo^4+^ on the catalyst surface reacted with Fe^3+^, and the regeneration of Fe^2+^ was then accelerated. After that, Fe^2+^ reacted with PMS through a series of redox reactions to generate free radicals. At the same time, HSO_5_^−^ produced by PMS hydrolysis could be reduced with a part of Fe^3+^ and Mo^6+^. Hydrogen ions in the solution could lead to protonation of Mo^6+^ generating Mo (VI) peroxy complex MoO(OH)(O_2_)_2_^−^ and further generate ^1^O_2_ [22].

## 4. Discussion

The above experimental results confirmed that the degradation efficiency of the O_3_/PMS/FeMoBC process was better than that of the O_3_ and O_3_/PMS process. In order to further explore the formation of intermediate products in the reaction process, it was analyzed by UPLC–MS/MS, molecular structural quantum chemical calculation and related research results. Finally, the toxicity of the above products was analyzed by ECOSAR and T.E.S.T. software to verify the toxicity change in the process in actual operation.

In the experiment, twelve kinds of intermediate products in the process of TC degradation were obtained by analyzing the original images of liquid mass spectrometry at different reaction times, and the mass-to-charge ratios were 445.16, 481.569, 477.152, 437.314, 419.458, 397.55, 338.393 and 148.151 respectively [29,30]. The results are shown in Table 3.

### 4.1. Construction of TC Molecular Model

In this process, firstly, the molecular structure of target compound TC was input by Chem3D v17.0 software (PerkinElmer, Inc., Waltham, MA, USA), and its initial configuration was successfully generated. Then, the structure was saved as a file in gif format and loaded in Gauss View 5.0 (Gaussian Inc., Wallingford, CT, USA) software environment. On this basis, the calculation parameters were set, density functional theory (DFT) was selected as the calculation method, and B3LYP hybrid functional was determined to be used for calculation. In B3LYP hybrid functional, B3 represents Becke’s three-parameter Lyapunov function, while LYP is Lee–Yang–Parr correlation functional. Then, the geometric optimization and vibration frequency analysis of the ground state structure of TC molecule were carried out, and the 6–31G (d, p) basis set was used for calculation.

**Figure 5:**
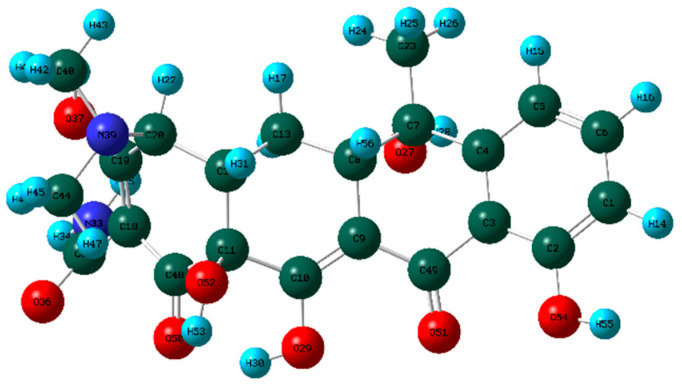
TC molecular model constructed by Gauss View.

After the geometric optimization of the TC molecule by DFT method, the stable structure of the TC molecule was obtained, and the result is shown in Figure 5. Subsequently, the highest occupied molecular orbital (HOMO) and the lowest empty molecular orbital (LUMO) of the TC molecule were studied in detail by using the software system of Gaussian09 (Gaussian Inc., Wallingford, CT, USA) and B3LYP/6-31G method. All geometric optimization and natural bond orbit (NBO) analyses were also completed by Gaussian 09 (Gaussian Inc., Wallingford, CT, USA) software.

The interaction between HOMO and LUMO can lead to the formation of excited states in the reaction; therefore, the molecular stability is often characterized by the energy difference (ΔE) between HOMO and LUMO. A smaller ΔE usually means that the compounds have higher chemical stability and lower reactivity [31]. While the larger ΔE indicates that the compound is chemically stable, it also means that the electron transfer process is more difficult, which may affect the ability of the compound to participate in chemical reactions, because electrons are not easy to transition from HOMO to LUMO. Therefore, when evaluating the chemical activity of compounds, it is necessary to comprehensively consider ΔE and other related factors. In quantum chemical calculation, if a point contributes more to HOMO (or LUMO), it may be the first choice for electrophilic (or nucleophilic) attacks [32]. The great contribution to HOMO means that compounds are more easily oxidized to cations, which makes them the target of electrophilic reagents. On the other hand, the great contribution to LUMO shows that molecules are more difficult to obtain electrons and more vulnerable to attack by nucleophiles.

### 4.2. Electron Cloud Distribution

By calculating and analyzing the molecular electron density (MED) diagram and electrostatic potential (ESP) diagram of TC, the electron distribution inside the organic molecule can be deeply understood. The MED diagram can reveal the local change in electron density in the molecule, while the ESP diagram shows the electrostatic potential on the surface of the molecule. Through the combination of the two, a comprehensive perspective can be provided to observe and understand the electronic structure of the TC molecule [33,34].

**Figure 6:**
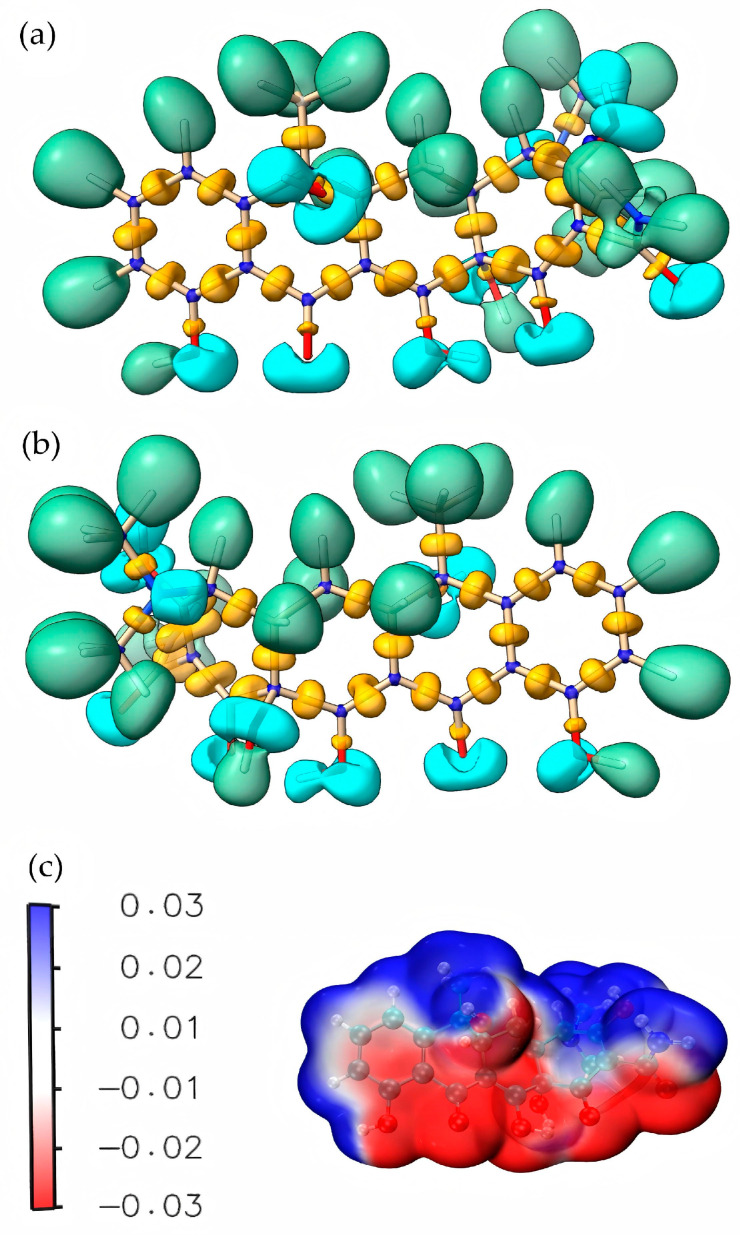
TC differential electron cloud distribution. (**a**) TC differential electron cloud density diagram I; (**b**) TC differential electron cloud density diagram II; (**c**) TC electrostatic potential diagram.

As seen in Figure 6, the analysis results shows that the amino (–NH_2_) region outside the benzene ring had a high electron density. This concentration of electron density indicated that this region had a strong electron cloud coverage, which made the N–C bond connected with it present an electron-rich state. Therefore, in the process of TC degradation, substances with a mass-to-charge ratio of 340.345 were generated by breaking the N–C bond of P4 and dehydration condensation reaction. In addition, the carbon–hydrogen bonds (C–H) in the benzene ring structure showed high electron density, which made them potential sites for reactive free radicals, which might lead to ring breakage. Meanwhile, C–H, which was directly connected with carbon atoms, also showed rich electronic characteristics, which made the hydrogen atoms connected with it more likely to have substitution or addition reactions with ^•^OH. Therefore, under the attack of ^•^OH, such C atoms were easy to form various substances with mass-to-charge ratios of 481.569, 437.314 and 338.393 (Path I). In addition, it could be observed that the C=O double bond (acyl group) in TC molecule was electron-deficient by superimposing the ESP diagram of the TC molecule on the MED diagram for analysis. Therefore, in the process of reaction, the reduction reaction produced a substance with a mass-to-charge ratio of 217.226, which could further react to form a substance with a mass-to-charge ratio of 134.117.

### 4.3. Frontier Molecular Orbital (FMO) Analysis

**Figure 7:**
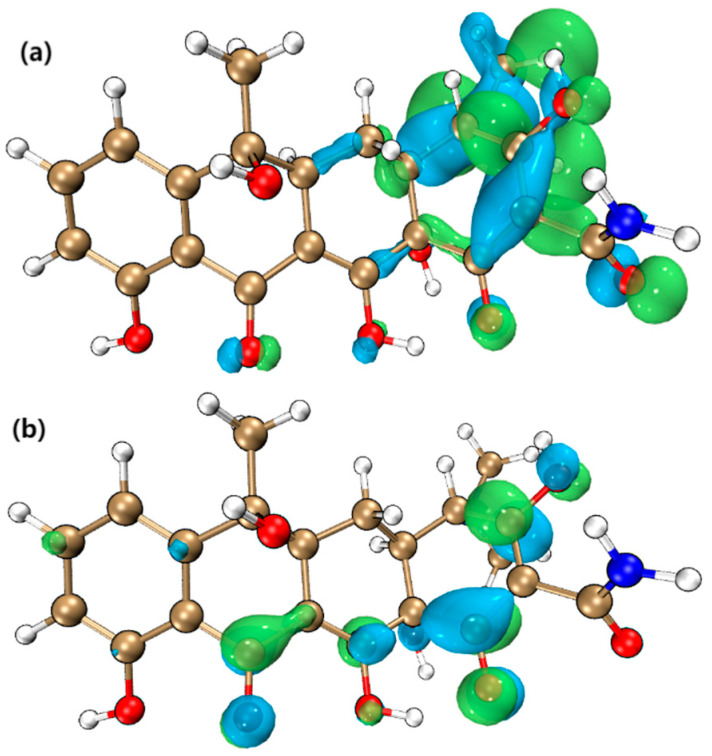
Frontier molecular orbital analysis of TC. (**a**) HOMO front orbit of TC; (**b**) LUMO front orbit of TC.

Figure 7 shows the distribution of HOMO and LUMO of TC molecules [35]. According to the FMO theory, these two orbitals play a decisive role in chemical reactions, and they represent the occupied orbitals with the highest energy and the unoccupied orbitals with the lowest energy [36]. Through the calculation of density functional theory (DFT), we knew that the HOMO energy of TC was −0.218390 Hartree (Ha), while the LUMO energy was −0.073063 Ha. These energy values reflected the relative electron affinity and electron providing ability of molecular orbitals.

Considering the reactivity of ^•^OH radicals with TC molecules, the HOMO energy of ^•^OH was −0.249425 Ha, which was close to LUMO energy of TC. This energy proximity meant that ^•^OH was more likely to transfer electrons with LUMO of TC, thus promoting the chemical reaction. Therefore, this showed that ^•^OH had high reactivity to TC molecules. In O_3_/PMS/FeMoBC system, the steady-state concentration of ^•^OH was significantly higher than that of ^•^O_2_^−^. This concentration difference further supported the possibility of the reaction between ^•^OH and TC. Because no intermediate product produced by the reaction between TC and ^•^O_2_^−^ was detected in this system, it was inferred that under this condition, the reactivity of ^•^O_2_^−^ was relatively low, or the reaction pathway it participated in was not the main degradation pathway.

### 4.4. Fukui Index Analysis

Fukui index (*f_k_*^0^) is a descriptor to quantify the reactivity of molecular active sites. Based on the molecular orbital theory, this index is defined by calculating the energy difference between HOMO and LUMO [37]. *F_k_*^0^ is defined as the arithmetic average of electron donating reactivity (*f_k_*^+^) and electron accepting reactivity (f*_k_*^−^) of an atom in a molecule, which can be expressed as Formula (2).

(2) $$f_{k}^{0}=\frac{f_{k}^{+}+f_{k}^{-}}{2}$$

Among which, *f_k_*^+^ reflects the tendency of atoms to lose electrons, while *f_k_*^−^ describes the tendency of atoms to gain electrons [38]. These two indexes are related to the frontier properties of molecular orbitals (HOMO and LUMO), respectively. In chemical reactions, atoms with higher *f_k_*^0^ value are more likely to be targeted by active radicals because they have higher reactivity in the process of electron transfer. Therefore, the Fukui index is an important tool to predict which atoms in molecules are more likely to participate in chemical reactions, especially in the reactions initiated by free radicals. For TC molecules, by calculating the Fukui index of each atom, those sites that were more vulnerable to active free radicals could be identified. The calculation results of TC molecular Fukui index are shown in Table 4.

As shown in Table 4, the *f_k_*^0^ value of each atom in the TC molecule was listed. By analyzing their Fukui indexes in detail, the research revealed the sensitivity of different atoms in the molecule to active radical attack. Among the C atoms, the C (19) atom in the benzene ring structure showed the highest *f_k_*^0^ value, which meant that the C (19) atom was one of the most likely reaction sites in the chemical reaction initiated by free radicals. In addition, several other carbon atoms on the benzene ring, such as C (10), C (48) and C (49), also show relatively high *f_k_*^0^ values. This indicated that these sites were also vulnerable to the attack of active free radicals to some extent, resulting in an intermediate product with *m*/*z* = 481.569. Substitution reaction is a common mechanism of free radical reaction, which usually leads to significant changes in molecular structure. In the process of TC degradation, ^•^OH radicals tended to attack C (19) atoms first and might generate molecular fragments with *m*/*z* = 477.152 through the substitution reaction of hydrogen atoms. In addition, the C (44) atom connected with the nitrogen atom N (39) also showed a high *f_k_*^0^ value, which indicated that the C–N bond in the TC molecule and the single bond between N (39) and C (44) might be easily broken during the chemical reaction. This breakage might lead to the recombination of the molecular structure and the formation of molecules with *m*/*z* = 419.458. It is worth noting that the Fukui index of oxygen atom O (51) was the highest among all atoms, which revealed that the chemical properties of carbon–oxygen double bonds in TC molecules were relatively unstable and vulnerable to attack [39]. This instability might be related to the electronic structure characteristics of double bonds and might also affect the behavior of molecules in chemical reactions and the formation of products [40].

### 4.5. Degradation Pathway Analysis

**Figure 8:**
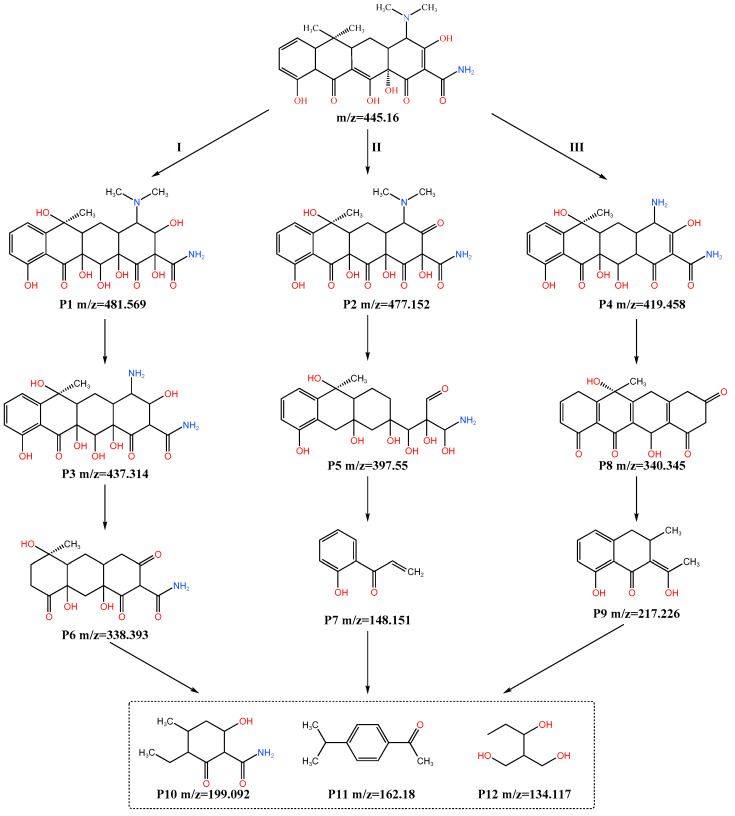
Proposed pathways of TC degradation in O_3_/PMS/FeMoBC.

The degradation process of TC molecules involved a series of complex chemical reactions, including the capture of hydrogen atoms, the substitution of functional groups and addition reactions, etc., which gradually led to the breaking of chemical bonds and the removal of functional groups [41,42]. With the progress of the reaction, TC molecules undergo ring-opening and structural decomposition, and finally transform into a variety of small molecular compounds [43]. In O_3_/PMS/FeMoBC system, the degradation pathway of TC is shown in Figure 8.

In degradation pathway I, ^•^OH first attacked TC molecules through addition reaction. According to the calculation results of electron cloud density distribution and Fukui index (*f_k_*^0^) of molecules, **C9** (*f_k_*^0^ = 0.04033), **C11** (*f_k_*^0^ = 0.03618) and **C18** (*f_k_*^0^ = 0.03677) atoms at the junction of benzene rings become the main targets of ^•^OH attacks because of their high reactivity, thus forming the intermediate **P1** (*m*/*z* =481.569 ). Subsequently, the methyl and amino groups on **N39** (*f_k_*^0^ = 0.04878) might be lost due to the attack of ^•^OH or ^1^O_2_, resulting in the formation of **P3** (*m*/*z* = 437.314) and **P6** (*m*/*z* = 338.393). **P6** was further subjected to cyclization, dihydroxylation and deamination to produce another intermediate **P11** (*m*/*z* = 162.18). This step showed that the structure of the TC molecule might change significantly during the degradation process. In addition, although **C8** and **C12** might also be attacked by ^•^OH, there was a big difference in their Fukui indexed compared with C9, so **P11** (*m*/*z* = 162.18) was the more important product in the process of ^•^OH or SO_4_^•−^ attacking benzene rings. This showed the effectiveness of the Fukui index in predicting the active sites of the reaction. In the subsequent stage of degradation pathway, ^•^OH might continue to attack other active sites on the benzene ring, which might lead to the formation of other intermediates such as **P12** (*m*/*z* = 134.117).

In pathway II, TC underwent hydroxylation, deoxidation with **O29** (*f_k_*^0^ = 0.0546), hydroxylation at **C9** (*f_k_*^0^ = 0.04033) and **C18** (*f_k_*^0^ = 0.03677) to form an intermediate **P2** (*m*/*z* = 477.152), followed by cyclization (**C20**, *f_k_*^0^ = 0.03811) to form **P5** (*m*/*z* = 397.55). **P5** underwent ring cleavage to form **P7** (*m*/*z* = 148.151). Subsequently, the intermediate **P7**–**P9** were further decomposed into three small molecular substances (**P10**~**P12**).

In pathway III, **P4** (*m*/*z* = 419.458) was produced by demethylation of TC at **N39** (*f_k_*^0^ = 0.04878). Subsequently, a byproduct **P8** (*m*/*z* = 340.345) was formed by deamination and a series of C–C bond recombination. It is worth noting that **P9** (*m*/*z* = 217.226) was produced by demethylation, right ring-opening reaction and gradual deamination. With the continuous degradation process, the intermediate products were gradually decomposed into simpler and lower molecular weight compounds. These compounds include H_2_O and CO_2_, which are signs of complete mineralization of organic compounds.

### 4.6. Toxicological Analysis of TC and Its Intermediate Products

In the field of environmental toxicology and drug development, quantitative structure–activity relationship (QSAR) model is widely used to predict the toxicity of compounds. In this experiment, the acute and chronic toxicity of TC and its intermediates were predicted by ECOSAR software. ECOSAR estimates the toxic impact of industrial chemicals on aquatic organisms in water bodies where the chemicals are released [44]. The ECOSAR program was used to predict the acute and chronic toxicity of OTC and its intermediate products on fish, green algae and daphnid. The toxicity data derived from ECOSAR were categorized by the Globally Harmonized System of Classification and Labelling of Chemicals (GHS), which delineates four toxicity classifications: not harmful (>100 mg/L), harmful (10–100 mg/L), toxic (1–10 mg/L), and highly toxic (<1 mg/L) [45]. In addition, T.E.S.T. software was used to predict the oral median lethal dose (**LD50**), developmental toxicity and Ames Test mutagenicity of TC and its intermediates in rats [46]. T.E.S.T, developed by US Environmental Protection Agency (US EPA), can be used to predict physicochemical, health toxicology and ecotoxicological properties. The software integrates QSAR models constructed by various algorithms and can give a comprehensive prediction result according to the applicability of substances in each model. These prediction results will help to evaluate the safety and environmental risks of TC and its degradation products.

#### 4.6.1. ECOSAR Analysis

These intermediates were formed during the degradation of TC molecules, and their structures and chemical properties might be different from those of the original TC molecules [47]. Therefore, it was very important to predict their toxicity for evaluating the environmental safety of the whole degradation process [48].

Table 5 shows that the oxidation products **P1** (*m*/*z* = 481.569), **P3** (*m*/*z* = 437.314) and **P6** (*m*/*z* = 338.393) of TC had high toxicity. However, according to the analysis of LC–MS/MS results, the concentration of **P6** (*m*/*z* = 338.393) was low. Among them, the **N39** (*f_k_^0^* = 0.04878) atoms on **P1** (*m*/*z* = 481.569) and **P3** (*m*/*z* = 437.314) were easily attacked by active oxidants, and **P11** (*m*/*z* = 162.18) and **P12** (*m*/*z* = 134.117) were formed after deamination and cyclization with the toxicity greatly reduced. In addition, in pathway II, the macromolecular substance **P2** (*m*/*z* = 477.152) was considered to be non–toxic at its initial stage. However, after a series of reactions such as ring cracking, the intermediate **P5** (*m*/*z* = 397.55) showed a significant increase in toxicity. This might be due to the structural change in **P5**, which led to the change in its biological activity. Subsequently, when **P5** (*m*/*z* = 397.55) was oxidized to **P7** (*m*/*z* = 148.151), its toxicity was significantly reduced. This decrease in toxicity might be due to the simpler structure of **P7**, which reduced its bioavailability and bioaccumulation potential, thus reducing the potential harm to organisms. In pathway III, the toxicity of intermediate products **P4** (*m*/*z* = 419.458), **P8** (*m*/*z* = 340.345) and **P9** (*m*/*z* = 217.226) gradually decreased after deamination, demethylation, ring opening and recombination of C–C bonds. These structural changes might lead to an increase in molecular polarity, the weakening of intermolecular force and a decrease in biological activity, thus reducing their toxicity to organisms. Among all the intermediate products, **P2** (*m*/*z* = 477.152), **P7** (*m*/*z* = 148.151), **P9** (*m*/*z* = 217.226) and **P11** (*m*/*z* = 162.18) were considered not toxic. These results showed that in the process of TC degradation, with the simplification of molecular structure and the removal of functional groups, the overall toxicity tended to decrease.

#### 4.6.2. T.E.S.T. Analysis

**Figure 9:**
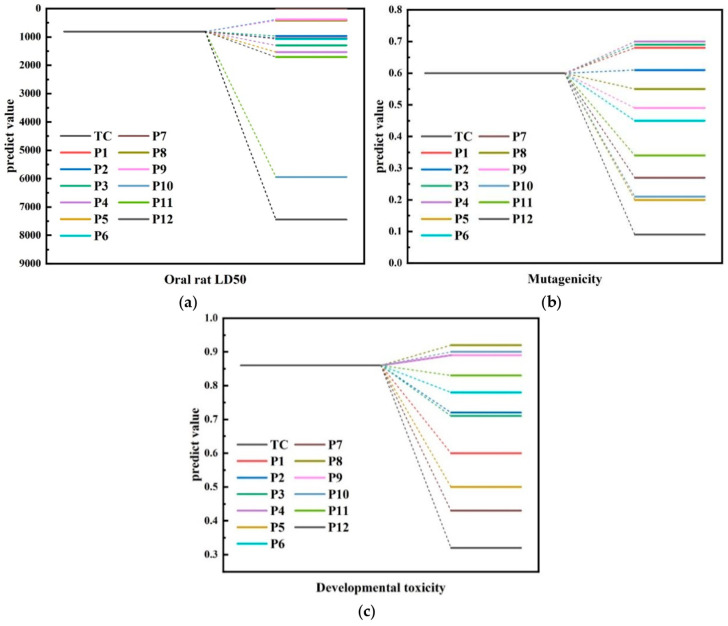
Intermediate product toxicity analysis of TC (T.E.S.T. prediction). (**a**) Oral rat **LD50**; (**b**) Mutagenicity; (**c**) Developmental toxicity.

According to the prediction results of T.E.S.T. software, the toxicology of TC and its 12 intermediates was deeply analyzed [46], and the results are shown in Figure 9 and Table 6. The predicted results included the information of oral median lethal dose (**LD50**), Ames mutagenicity and developmental toxicity in rats, which provided an important basis for evaluating the environmental and health risks of the compounds.

As shown in Figure 9a, in the prediction of **LD50**, three intermediate products, **P5** (*m*/*z* = 397.55), **P8** (*m*/*z* = 340.345) and **P9** (*m*/*z* = 217.226), were classified as moderate toxicity (Grade 3), among which **P9** was the most toxic. **P10** (*m*/*z* = 199.092) and **P12** (*m*/*z* = 134.117) were predicted to be slightly toxic (grade 5). Except for the missing data of **P7**, other intermediate products including TC were predicted to be low toxic (Grade 4). This result showed that with the degradation of TC, some intermediates derived from TC might show high toxicity, but the overall **LD50** value showed a downward trend with the degradation process.

In Ames mutagenicity prediction, as shown in Figure 9b, only **P1** (*m*/*z* = 481.569), **P2** (*m*/*z* = 477.152), **P3** (*m*/*z* = 437.314), **P4** (*m*/*z* = 419.458) and **P8** (*m*/*z* = 340.345) showed positive results, which meant that these compounds might have the ability to induce colony growth in Salmonella typhimurium strains. However, other compounds showed negative results, indicating that they did not have the ability to induce mutagenesis.

As shown in Figure 9c, for the prediction of developmental toxicity, **P7** (*m*/*z* = 148.151) and **P12** (*m*/*z* = 134.117) were predicted to be non-toxic, while the other 10 compounds, including TC, were predicted to be toxic, which indicated that they might have potential adverse effects on the biological development process.

## 5. Conclusions and Future Directions

(1) Among the three O_3_ processes, the O_3_/PMS/FeMoBC process achieved the highest mineralization degree, while the O_3_ process showed the lowest mineralization effect. This showed that the synergistic effect of various active oxidation substances in O_3_/PMS/FeMoBC system significantly improved its mineralization ability to organic matter. In contrast, when ozone only was used as oxidant, the degradation effect of organic matter was not ideal due to the limitation of its oxidation ability.
(2) The raw water experiment showed that the raw water sample had a certain influence on the degradation efficiency of O_3_/PMS/FeMoBC, mainly because there were many types of inorganic salts and other complex organic substances in the raw water sample. These complex fluorescent substances interfered with electron transfer in the system and consumed some active oxidizing substances. However, the O_3_/PMS/FeMoBC process still had a 98.8% degradation rate of TC in raw water. After 60 min of treatment, the fluorescent substances in the water almost completely disappeared.
(3) In the cyclic catalytic experiment, the k_obs_ value of the material decreased after repeated use, indicating that the catalytic activity of the catalyst decreased, but the material still had certain activity after five cyclic experiments.
(4) Based on the experimental results of LC–MS/MS and the quantum chemical calculation of molecular structure, the degradation path of TC was inferred. According to the theoretical calculation of DFT of Gaussian09 software, the main attack sites of TC molecular degradation were inferred. Twelve kinds of fragments with different mass-to-nucleus ratios (*m*/*z*) could be detected according to the scanning mass spectrum and the data of the intermediate products, and it could be clearly determined that the decomposition of TC main parent ions mainly occurred at C, N, O and other heteroatoms. At the initial stage of degradation, the hydrogen abstraction and substitution reactions were mainly initiated by ^•^OH, accompanied by deamination. The hydroxyl groups of alcohols connected to benzene rings were easily converted into aldehyde groups during oxidation, but these aldehyde groups would be lost in subsequent reactions. With the deepening of degradation, the benzene ring structure would also undergo ring opening and eventually be transformed into a series of small molecular products.
(5) In the toxicological analysis of TC degradation, the results of ECOSAR showed that with the gradual ring-opening and bond-breaking of TC to form small molecular compounds, its overall toxicity showed a downward trend. The analysis results of T.E.S.T. software showed that the **LD50** values of intermediate products were generally low, indicating that the acute toxicity of these products was relatively small. Nevertheless, **P1**, **P2**, **P3**, **P4** and **P8** showed positive reactions in the Ames mutation test, suggesting that these specific intermediates might have genotoxicity. The prediction of developmental toxicity revealed that TC and some of its intermediate products might have a potential impact on the development of organisms, which needed to be paid attention to in environmental risk assessment.
(6) Based on the above results, this study demonstrated the feasibility of the O_3_/PMS/FeMoBC process to degrade TC in a water environment, which provided a new idea for the treatment of high-concentrated organic wastewater. However, the discussion between the theoretical analysis of intermediate products formation and the role of catalysts in the reaction system is not still known. It might be realized through more advanced characterization means or a detailed theoretical analysis process. Therefore, the full combination of theoretical analysis and experimental means is a valuable future research direction.

## Figures and Tables

**Table 1:** Results of water quality analysis of water samples at the fine grating of the Dongnan Wastewater Treatment Plant.

Water Quality Index	Before Treatment (Including TC)	After Treatment
chemical oxygen demand (COD)	204.68 mg/L	45.61 mg/L
total nitrogen (TN)	39.68 mg/L	25.06 mg/L
ammonia nitrogen (NH_3_–N)	33.93 mg/L	21.24 mg/L
total phosphorus (TP)	5.59 mg/L	4.55 mg/L
pH	7.41	6.48

**Table 2:** Three-dimensional fluorescence partitioning method.

Zone	Organic Matter Type	Fluorescence Peak Position (Ex/Em)
I	Simple aromatic protein I (Tyrosine)	240–250 nm/280–330 nm
II	Simple aromatic protein II (Tryptophan)	240–250 nm/330–380 nm
III	Aromatic protein or phenols (Fulvic acid)	240–250 nm/380–550 nm
IV	Aromatic protein or phenols (Fulvic acid)	250–400 nm/300–380 nm
V	Humic acids	250–400 nm/380–550 nm

**Table 3:** TC intermediate statistics (identified by UPLC–MS/MS).

Compound ID	Experimental *m*/*z*	Molecular Formula	Proposed Chemical Structure
TC	445.16	C_22_H_24_N_2_O_8_	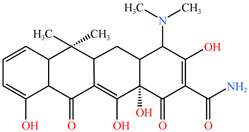
**P1**	481.569	C_22_H_28_O_10_N_2_	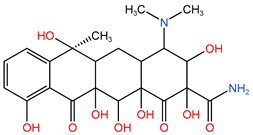
**P2**	477.152	C_22_H_24_O_10_N_2_	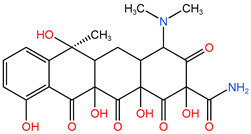
**P3**	437.314	C_20_H_24_O_9_N_2_	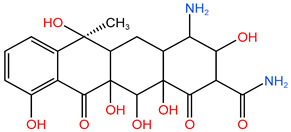
**P4**	419.458	C_20_H_22_O_8_N_2_	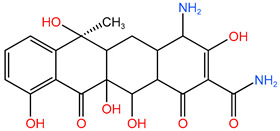
**P5**	397.55	C_19_H_27_O_8_N	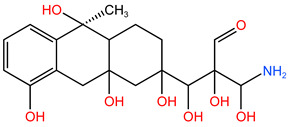
**P6**	338.393	C_16_H_21_O_7_N	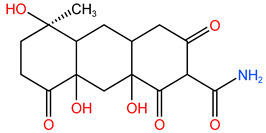
**P7**	148.151	C_9_H_8_O_2_	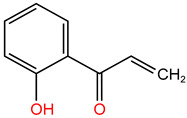
**P8**	340.345	C_19_H_16_O_6_	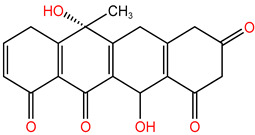
**P9**	217.226	C_13_H_14_O_3_	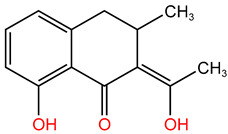
**P10**	199.092	C_10_H_17_O_3_N	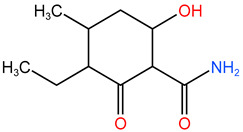
**P11**	162.18	C_11_H_14_O	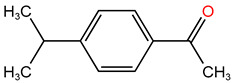
**P12**	134.117	C_6_H_14_O_3_	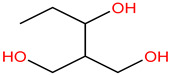

**Table 4:** Fukui index of TC molecule.

Atom	*f_k_* ^+^	*f_k_* ^−^	*f_k_* ^0^	Atom	*f_k_* ^+^	*f_k_* ^−^	*f_k_* ^0^
1 (C)	0.03019	0.03378	0.03198	29 (O)	0.01981	0.0335	0.02665
2 (C)	0.0346	0.02794	0.03127	30 (H)	0.00286	0.003	0.00293
3 (C)	0.01293	0.01309	0.01301	31 (H)	0.00087	0.0027	0.00178
4 (C)	0.02114	0.01392	0.01753	32 (C)	0.02732	0.01332	0.02032
5 (C)	0.02513	0.03568	0.0304	33 (N)	0.01131	0.02909	0.0202
6 (C)	0.04273	0.02554	0.03414	34 (H)	0.00176	0.00245	0.0021
7 (C)	0.00683	0.01317	0.01	35 (H)	0.00182	0.00248	0.00215
8 (C)	0.00495	0.01405	0.0095	36 (O)	0.01658	0.06473	0.04065
9 (C)	0.03007	0.0506	0.04033	37 (O)	0.03648	0.01495	0.02571
10 (C)	0.05907	0.03024	0.04465	38 (H)	0.00439	0.00121	0.0028
11 (C)	0.04058	0.03177	0.03618	39 (N)	0.01113	0.08643	0.04878
12 (C)	0.00372	0.0102	0.00696	40 (C)	0.0021	0.01255	0.00733
13 (C)	0.00279	0.00891	0.00585	41 (H)	0.00086	0.00808	0.00447
14 (H)	0.00325	0.00273	0.00299	42 (H)	0.00076	0.00278	0.00177
15 (H)	0.00254	0.00317	0.00285	43 (H)	0.00065	0.00183	0.00124
16 (H)	0.00454	0.00227	0.00341	44 (C)	0.00286	0.0132	0.00803
17 (H)	0.00074	0.002	0.00137	45 (H)	0.00053	0.00262	0.00157
18 (C)	0.03936	0.03418	0.03677	46 (H)	0.00126	0.00824	0.00475
19 (C)	0.0977	0.01822	0.05796	47 (H)	0.00137	0.00254	0.00195
20 (C)	0.03522	0.04101	0.03811	48 (C)	0.10183	0.01352	0.05768
21 (H)	0.0017	0.0023	0.002	49 (C)	0.0729	0.021	0.04695
22 (H)	0.00377	0.00169	0.00273	50 (O)	0.07231	0.04059	0.05645
23 (C)	0.0013	0.00738	0.00434	51 (O)	0.06216	0.08442	0.07329
24 (H)	0.0003	0.00121	0.00076	52 (O)	0.01558	0.02516	0.02037
25 (H)	0.00065	0.00168	0.00117	53 (H)	0.00216	0.00193	0.00204
26 (H)	0.00056	0.00131	0.00094	54 (O)	0.01006	0.03036	0.02021
27 (O)	0.00607	0.03957	0.02282	55 (H)	0.00174	0.00201	0.00187
28 (H)	0.002	0.00264	0.00232	56 (H)	0.0022	0.00515	0.00367

**Table 5:** Toxicity of TC and its intermediates (predicted by ECOSAR).

Compound	Acute Toxicity (mg/L)			Chronic Toxicity (ChV) (mg/L)		
	Fish LC50 (96 h)	Daphnid LC50 (48 h)	Green Algae EC50 (96 h)	Fish LC50	Daphnid LC50	Green Algae EC50
TC	13,100	1060	1890	2490	59.9	474
**P1**	66,500	4780	10,700	18,100	240	2470
**P2**	56.70	7.02	5.38	2.950	0.597	1.83
**P3**	321,000	20,300	58,800	129,000	901	12400
**P4**	49,200	3580	7850	12,900	182	1830
**P5**	3480	4900	1740	4700	23	334
**P6**	772,000	308,000	53,100	49,700	11200	6320
**P7**	62.10	36.40	30.70	6.290	3.850	8.58
**P8**	9030	4390	1720	735	278	319
**P9**	6.99	4.59	6.22	0.811	0.670	2.25
**P10**	14,800	6880	2230	1140	383	373
**P11**	12.90	8.12	9.31	1.420	1.060	3.08
**P12**	5020	2410	885	402	146	159

**Table 6:** Toxicity of TC and its intermediates (predicted by T.E.S.T.).

Compound	Oral Rat LD50	Ames Mutagenicity		Developmental Toxicity	
	Predicted Value (mg/kg)	Predicted Value	Predicted Result	Developmental Toxicity Value	Developmental Toxicity Result
TC	806.96	0.60	Positive	0.86	toxicant
**P1**	1042.49	0.68	Positive	0.60	toxicant
**P2**	964.51	0.61	Positive	0.72	toxicant
**P3**	1299.00	0.69	Positive	0.71	toxicant
**P4**	1531.92	0.70	Positive	0.89	toxicant
**P5**	414.69	0.20	Negative	0.50	toxicant
**P6**	1068.17	0.45	Negative	0.78	toxicant
**P7**	N/A	0.27	Negative	0.43	non-toxicant
**P8**	419.99	0.55	Positive	0.92	toxicant
**P9**	381.32	0.49	Negative	0.89	toxicant
**P10**	5941.00	0.21	Negative	0.90	toxicant
**P11**	1711.39	0.34	Negative	0.83	toxicant
**P12**	7445.82	0.09	Negative	0.32	non-toxicant

## Data Availability

Data are contained within the article.
